# Auditory word recognition of verbs: Effects of verb argument structure on referent identification

**DOI:** 10.1371/journal.pone.0188728

**Published:** 2017-12-05

**Authors:** Mònica Sanz-Torrent, Llorenç Andreu, Javier Rodriguez Ferreiro, Marta Coll-Florit, John C. Trueswell

**Affiliations:** 1 Grup de Recerca Cognitiva i Llenguatge (GRECIL), Departament de Cognició, Desenvolupament i Psicologia de l’Educació, Universitat de Barcelona, Barcelona, Spain; 2 Grup de Recerca Cognitiva i Llenguatge (GRECIL), Estudis de Psicologia i Ciències de l’Educació, Universitat Oberta de Catalunya, Barcelona, Spain; 3 Estudis d’Arts i Humanitats, Universitat Oberta de Catalunya, Barcelona, Spain; 4 Department of Psychology, University of Pennsylvania, Philadelphia, Pennsylvania, United States of America; Boston Children’s Hospital / Harvard Medical School, UNITED STATES

## Abstract

Word recognition includes the activation of a range of syntactic and semantic knowledge that is relevant to language interpretation and reference. Here we explored whether or not the number of arguments a verb takes impinges negatively on verb processing time. In this study, three experiments compared the dynamics of spoken word recognition for verbs with different preferred argument structure. Listeners’ eye movements were recorded as they searched an array of pictures in response to hearing a verb. Results were similar in all the experiments. The time to identify the referent increased as a function of the number of arguments, above and beyond any effects of label appropriateness (and other controlled variables, such as letter, phoneme and syllable length, phonological neighborhood, oral and written lexical frequencies, imageability and rated age of acquisition). The findings indicate that the number of arguments a verb takes, influences referent identification during spoken word recognition. Representational complexity and amount of information generated by the lexical item that aids target identification are discussed as possible sources of this finding.

## Introduction

A great deal of experimental evidence supports the idea that word recognition includes the activation of a range of syntactic and semantic knowledge that is relevant to language interpretation and reference (e.g., [1–4]). This information is believed to include syntactic category information (e.g., noun, verb, adjective), combinatory syntactic information (e.g., the number and types of syntactic complements the word assigns), non-combinatory semantic information (animacy, etc.) and combinatory semantic information (the number and types of semantic entities, or arguments/roles). It is also believed that this information is activated in real-time, as a function of the frequency and contextual relevance of the word (e.g., [5–9]). Moreover, neuroscientific evidence exists to support the idea that this information is functionally organized in the brain and activated in response to the recognition of lexical items, with the evidence coming from event-related potentials (e.g., [10–13]), functional Magnetic Imaging, fMRI [14–15] and lexical processing dissociations related to brain damage (e.g., [16–29]).

Given this evidence, one might expect that the sheer amount of information that a word activates would impinge negatively on its processing time. Yet, this expectation is not always confirmed in studies of language processing. For example, some reading time data suggests that the processing time of a verb is not a function of its argument structure or semantic complexity, (e.g., [30–32]), whereas other reading data seem to implicate that lexical semantic complexity influences reading times (e.g., [33–34]). In particular, Inhoff [30] found that factive and nonfactive verbs did not receive different fixation times. Rayner and Duffy [31] showed that verb complexity does not affect lexical access time in that causative, factive and negative verbs did not influence fixation times. Schmauder [32] was unable to find evidence that the number of semantic arguments influences the ease of processing during language comprehension. In contrast, Gennari and Poeppel [33] found that eventive verbs showed longer processing times than stative verbs. Moreover, McElree et al. [34] showed that reading times were longer for complements that required type-shifting than for complements that directly matched the semantic restrictions of the matrix verb. Likewise, some cross-modal lexical decision tasks that were designed to tap processing difficulty have found positive effects of verb argument complexity (e.g., [35–37]), whereas others have not been able to find these effects [38–39]. In particular, Shapiro and collaborators [35–37]) found in different studies that verb’s representational complexity (syntactic subcategorization and argument structure) affects real-time sentence processing. On the other hand, Schmauder and collaborators [38–39] did not find this effect in cross-modal lexical decision and monosyllabic secondary lexical decision tasks.

Perhaps these inconsistent results relating lexical processing time to representational complexity, in the face of overwhelming evidence that such lexical information is indeed computed in response to encountering a word, are evidence favouring cost-free parallel activation of lexical information (e.g., [40–43]). From this perspective, lexical processing time is not related to representational complexity per se but may instead be related to how well it informs the task at hand. For example, verb information would have a greater impact on reading times in a sentence if it generated structural ambiguity related to the meaning. On the other hand, there are other theories that appeal to semantic and syntactic complexity on its own to explain processing times (e.g., [33, 44]). This account assumes that the more complex a word is, the more processing time it takes. For example, Gennari and Poeppel [33] used a lexical decision task and a self-paced reading study to analyze the processing times of eventive verbs, which denote causally structured events, and stative verbs, which denote facts without causal structure. As they expected, the conceptually more complex eventive verbs took longer to process than stative verbs in both tasks.

In our study, we seek to analyse in greater detail if lexical processing time increases as a function of representational complexity. Concretely, we aim to study if hearing a verb in isolation and looking for its possible referent is influenced by verb argument complexity. We operationalize “verb argument complexity” as the number of arguments that a verb takes. Jackendoff [45] states that the verb defines the number of semantic arguments, and determines which of these arguments have to be expressed in the syntactic structure of the sentence in which the verb is embedded. For example, the verb “to hit” must have two arguments (the “agent” (the hitter) who executes the action and the “patient” (the person or thing being hit, who suffers from the action). In contrast, the verb “to give” has three arguments (“agent”, “theme” and “recipient”). Verbs can take one, two, and three arguments. These three types of verbs show an incremental semantic and syntactic complexity. These differences will allow us to analyse if lexical processing time will also increase as a function of the number of verb arguments. Because the number of syntactic complements and the number of semantic arguments a verb can take are highly related we cannot in this study distinguish between syntactic and semantic complexity and so simply use the term *verb argument complexity* to refer to both.

The experiments below used the *visual world paradigm* [46, 47] to study this issue. In this paradigm participants’ eye movements are recorded as they hear words that refer to visually present referents. For example, Allopenna et al. [5] recorded listener’s eye movements to a visual display containing a target object (e.g., a beaker), a rhyme competitor (e.g., a speaker), and an unrelated competitor (e.g., a carriage). Participants followed a spoken instruction to move one of the objects with a mouse. Initial fixations were equally likely to be directed to the target and cohort but after the disambiguating point, looks went towards the target. Moreover, after this point, the rhyme competitor received more fixations than unrelated items. Subsequent work revealed that word recognition is affected by frequency [48], neighborhood density [49] and coarticulatory mismatch [50]. These findings showed that eye movements in this task can provide a real-time window on the dynamics of lexical activation.

To date there exists only one visual world study that examined how word recognition is related to verb argument complexity but it was conducted only with children (see [51]). Andreu, Sanz-Torrent and Guàrdia-Olmos [51] compared the dynamics of spoken word recognition for nouns and verbs with different argument structure preferences, in Spanish-speaking children with and without Specific Language Impairment. All the groups recognized nouns faster than verbs and recognized one-argument verbs faster than two- and three-argument verbs—although all effects occurred later, after word offset. It was also observed that children with SLI were slower than their controls, especially in the recognition of three-argument verbs.

Using the same method, the present study sought to examine whether adults’ speed to identify words vary with different verb argument complexity in auditory single word recognition. This will provide us with more accurate results than the previous methods such as cross-modal lexical decision task or self-paced reading because the *visual world paradigm* provides not only a time measure that participants take to activate a word but also the temporal dynamics moment-by-moment associated with the activation of lexical semantic information.

Previous studies reviewed above showed inconsistent results relating lexical processing time to verb argument complexity (e.g., [30–34]). On the one hand, there are a group of studies that have found that processing time is not related to the number of verb arguments [35–37]). If this is the case, we won’t find differences on word recognition as the verb arguments increases. On the other hand, if processing time is affected by argument complexity ([as 32]), our results will show that the latency times will be longer as the number of verb arguments increases.

In this work, we report three experiments comparing the dynamics of spoken word recognition for verbs with different argument complexity. In experiment 1, we recorded listeners’ eye movements as they searched an array of pictures in response to hearing a verb. The target verb differed in terms of the number of arguments it takes (1, 2 or 3 arguments). In experiment 2, participants had the same task but also had to indicate via a button press exactly when they had heard the target word in the input stream. Finally, in experiment 3, we controlled more variables that could in principle affect the auditory word recognition. We extended the sample and increased the number of verb stimuli.

## Experiment 1

### Method

This study was approved by the Ethics Committee of the Universitat Oberta de Catalunya.

### Participants

Thirty-one native Spanish speakers participated in the experiment (16 females and 15 males). All participants were born in Spain and studied primary and secondary school in Spain. They were students or junior faculty at various Universities in the Barcelona area. All participants either had uncorrected vision or wore soft contact lenses or eyeglasses. They gave their written informed consent for participation in this study.

### Stimuli

We used the same stimuli as in Andreu, Sanz-Torrent and Guàrdia-Olmos [51]. Eighteen verbs (six one-argument, six two-argument and six three-argument verbs) were used as target words. Moreover, 18 nouns were used as a target for filler stimuli (see S1 Appendix). Both sets of words (verbs and nouns) were selected following the same criteria: they had to be very common words and easily recognizable from visual stimuli (for example, events denoted by the verbs *walk*, *open* or *tie* are easily recognizable but others like those denoted by the verbs *think*, *love*, etc. not). A preliminary list of words was first created and then only those words that received relatively high imageablity ratings were selected. All the words were matched for number of syllables such that there were the same number of monosyllabic (one), disyllabic (thirteen) and trisyllabic words (four). Verbs with different argument structure and the nouns had the same mean syllable length of 2.16. In addition, for both verbs and nouns we controlled the frequency of written Spanish (using the LEXESP corpus [52]). At the time experiment 1 was conducted we had no access to Spanish oral lexical frequency data, but given that the Spanish is a language with a very shallow orthographic system, we used written frequency. In addition, we also controlled the imageability from published rating norms [53]; the label appropriateness from a separate group of 32 adults ratings (values 1–7) and the mean age of first production using the program FREQ of the CLAN (CHILDES project [54]) from Serra-Solé and Vila corpus and own authors’ database which includes monthly speech transcriptions of 13 children from ages 1 to 4 approximately.

Table 1 shows that the three verb subsets did not differ significantly on any of the variables, although there was a marginally significant uncorrected pairwise comparison in the imageability between one-argument and two-argument verbs [t(10) = 2.07, p = 0.07]. Table 2 also shows that, as expected, verbs did not differ from filler nouns in any way except for imageability and label appropriateness. (see, e.g., Gillette, Gleitman, Gleitman and Lederer [55] for a similar effect in English).

**Table 1:** Mean properties of verb classes (SD) and (range) in parentheses. F-Ratios reflect effect of verb class.

	One-argument N = 6 Mean (S.D.) (range)	Two-argument N = 6 Mean (S.D.) (range)	Three-argument (N = 6) Mean (S.D.) (range)	F-Ratio Test
*Frequency*	41.52 (26.68) (17.14–84.82)	38.63 (35.04) (1.79–102.5)	54.11 (96.21) (4.46–249.11)	F(2,15) = 0,065 p = 0.938
*Age of first production*	21.33 (5.32) (18–30)	28.17 (3.60) (21–30)	21.17 (4.35) (18–29)	F(2,15) = 3.706 p = 0.123
*Imageability*	5.56 (0.65) (4.9–6.38)	4.12 (1.58) (1.07–5.42)	4.94 (0.74) (3.64–5.8)	F(2,15) = 2.72 p = 0.10
*Label Appropriateness*	4.63 (0.92) (3.52–5.93)	4.18 (0.50) (3.35–4.68)	4.29 (0.83) (3.61–5.39)	F(2,15) = 0.57 p = 0.58

Note: Frequency: word frequency per million; age of first production: months; imageability: mean subjective imageability in a 1–7 scale; label appropriateness: subjective ratings in a 1–7 scale.

**Table 2:** Mean properties of target verbs and filler nouns (SD) and (range) in parentheses. **F-Ratios reflect effect** of syntactic category.

	Target verbs N = 18 Mean (S.D.) (range)	Filler nouns N = 18 Mean (S.D.) (range)	F-Ratio Test
*Frequency*	44.75 (57.80) (1.79–249.11)	47.84 (49.46) (4.64–172.14)	F(1,34) = 0.024 p = 0.880
*Age of first production*	23.55 (5.38) (18–30)	22.89 (5.92) (18–30)	F(1,34) = 0,108 p = 0.746
*Imageability*	4.87 (1.18) (1.07–6.38)	6.29 (0.34) (5.77–7.00)	F(1,34) = 24.17, p<0.001
*Label Appropriateness*	4.37 (0.73) (3.35–5.93)	6.26 (0.67) (4.32–6.97)	F(1,34) = 63.08 p<0.001

Note: Frequency: word frequency per million; age of first production: months; imageability: mean subjective imageability in a 1–7 scale; label appropriateness: subjective ratings in a 1–7 scale.

In sum, within the set of verbs, verbs with one, two or three arguments did not differ from each other in terms of how imageable they were or how appropriate they were as labels for their pictures. On the other hand, filler nouns differed from target verbs in that they were more imageable and more appropriate as labels for their corresponding pictures.

As described in Andreu, Sanz-Torrent and Guàrdia-Olmos [51], the 36 words (18 verbs and 18 nouns) were paired with a picture depicting the action or object. Each target picture was then paired with three additional pictures such that the resulting set of four images always included two event images and two object images. Target verbs had one event competitor and two object distracters and filler stimuli had one object competitor and two event distracters. The preferred names for competitor and distracter pictures were similar in frequency to the target names (using the LEXESP corpus [52]). In addition, the onset phoneme of each target word always differed from the onset phoneme of the words for the competitor picture and the two distracter pictures, so to avoid auditory cohort competitor effects (see Allopenna et al [5]).

**Fig 1:**
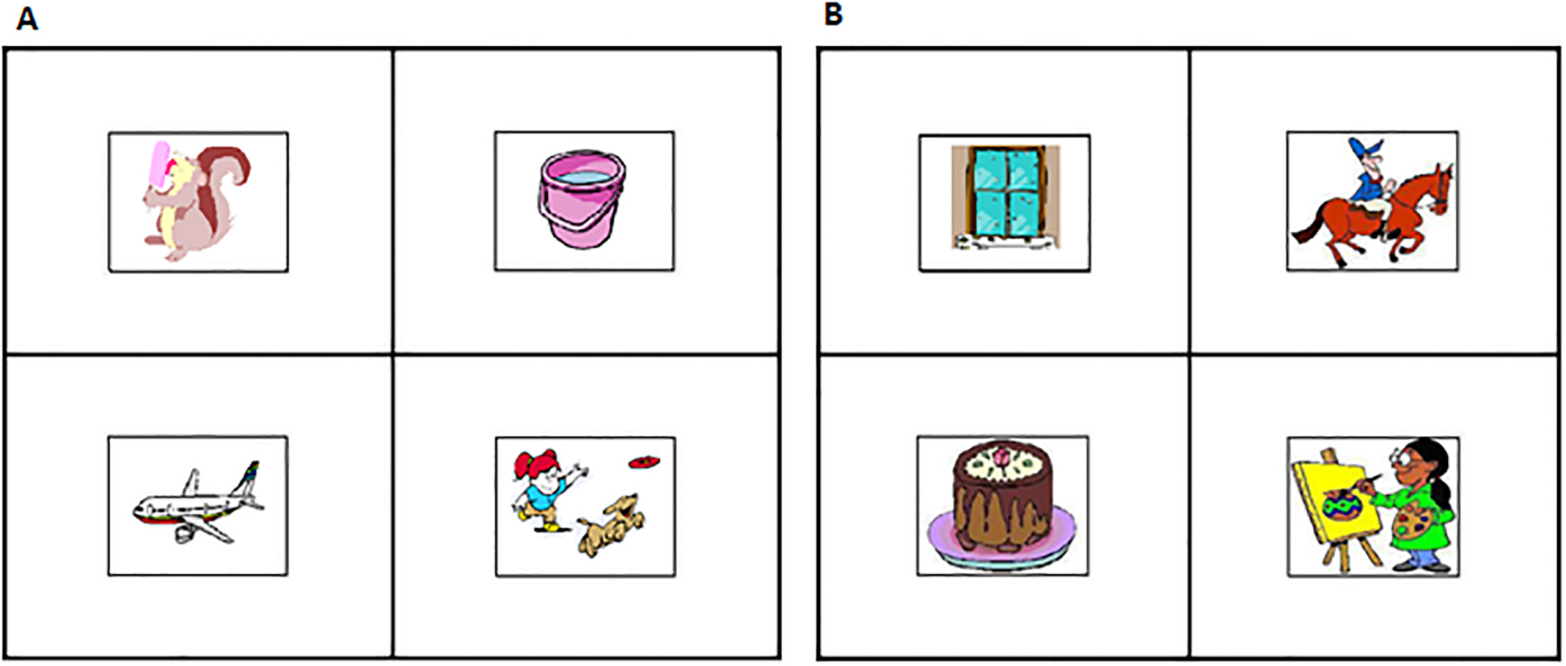
Stimuli example. (A) Target verb: To lick (one-argument verb); competitor: To launch (three-argument verb); distracters: plane and cauldron. (B) Filler stimulus: Target Noun: cake; competitor: window; distracters: To ride (two-argument verb) and to paint (two-argument verb).

Target words were recorded by a male native Spanish speaker and sampled at 44,100 Hz. Each trial image consisted of four pictures each placed within four quadrants on the computer screen (see Fig 1). The background was white and had two black lines, one vertical, one horizontal, were used to divide the four quadrants. The position of the target picture, the competitors and distracters were randomized in these four quadrants. Moreover, the number of arguments involved in target and competitor event pictures were balanced across conditions. In particular, for the six target items within each condition, two always appeared with a one-argument competitor, two appeared with a two-argument competitor and two appeared with a three-argument competitor. Finally, we carefully selected distracter pictures so that their appearance or similarities in form, function or color were not similar to the targets.

The audio and the visual image for each item were merged together in a video file lasting 4000 ms, using VirtualDubMod software. In each video, the onset of the spoken word coincided with the onset of the visual stimuli. The spoken word finished around 1000ms from image onset.

### Procedure

Participants were seated approximately 22” in front of a *Tobii T120* eye tracker with an integrated 17” TFT monitor. *Tobii Studio* Software was used to present the stimuli, and collect the eye tracking data. Stimuli videos were 800 x 600 pixels in size and centered on the screen, which was set to 1024 x 768 pixels. The visual angle of each object subtended approximately 13 degrees, well above the 0.5 degree accuracy of the eye tracker. All audio was played over a mono channel split to two loudspeakers positioned on either side of the viewing monitor. Eye position was sampled at 120Hz (i.e., at 8.333 ms intervals).

A nine point calibration procedure was carried out at the beginning of the experiment. The *Tobii Studio* Software automatically validates calibrations and the experimenter could, if required, repeat the calibration process if validation was poor. Calibration took approximately 20 s. Participants were instructed that for each trial they would see a set of four pictures and hear a single word spoken aloud. Their task was to find the picture mentioned, and then continue looking at the picture until the video disappeared. There were two practice trials before the experimental task (one with a verb target and one with a noun target) to acquaint the participant with the flow of events. The test videos were presented in random order in two blocks. Each block contained eighteen different words (nine target verbs, three of each verb type and nine fillers in which the noun was the target). All the participants were given both blocks. Between each trial, participants were presented with a crosshair centered in the middle of the screen (which they had been instructed to fixate). This position was equidistant from each quadrant and corresponded to the intersection of the two lines that divided the four quadrants. The crosshair was displayed for 2000 ms.

### Analysis

For each target picture, there was a pre-defined area of interest that consisted of a rectangle surrounding the picture (see Fig 1). The horizontal and vertical eye position data was then used to determine looks to the target picture (See S2 Appendix). A value of one was given to every eye-tracking sample that fell within the target region; otherwise it was given a zero (looks to other areas, off the screen or track loss). Then, for each participant on each trial, the proportion of looks to the target was calculated during two time windows following Andreu, Sanz-Torrent and Guàrdia-Olmos [51]. The first window began 200 ms after the onset of the spoken word (and video) and lasted until the end of the word, which was always 1000 ms. A 200 ms offset was used because the minimum latency to plan and launch a saccade is estimated to be between 150 and 180 ms in simple tasks [56–58]. As such, 200 ms after word onset is approximately the earliest point at which one expects to see looks driven by the acoustic information. The second time window corresponded to 1000–2000 ms, which was a one second interval after the word was uttered.

Finally, trials with more than 33% track loss were excluded. The mean percent of track loss was 2.7% resulting in the need to drop three trials.

### Results

**Fig 2:**
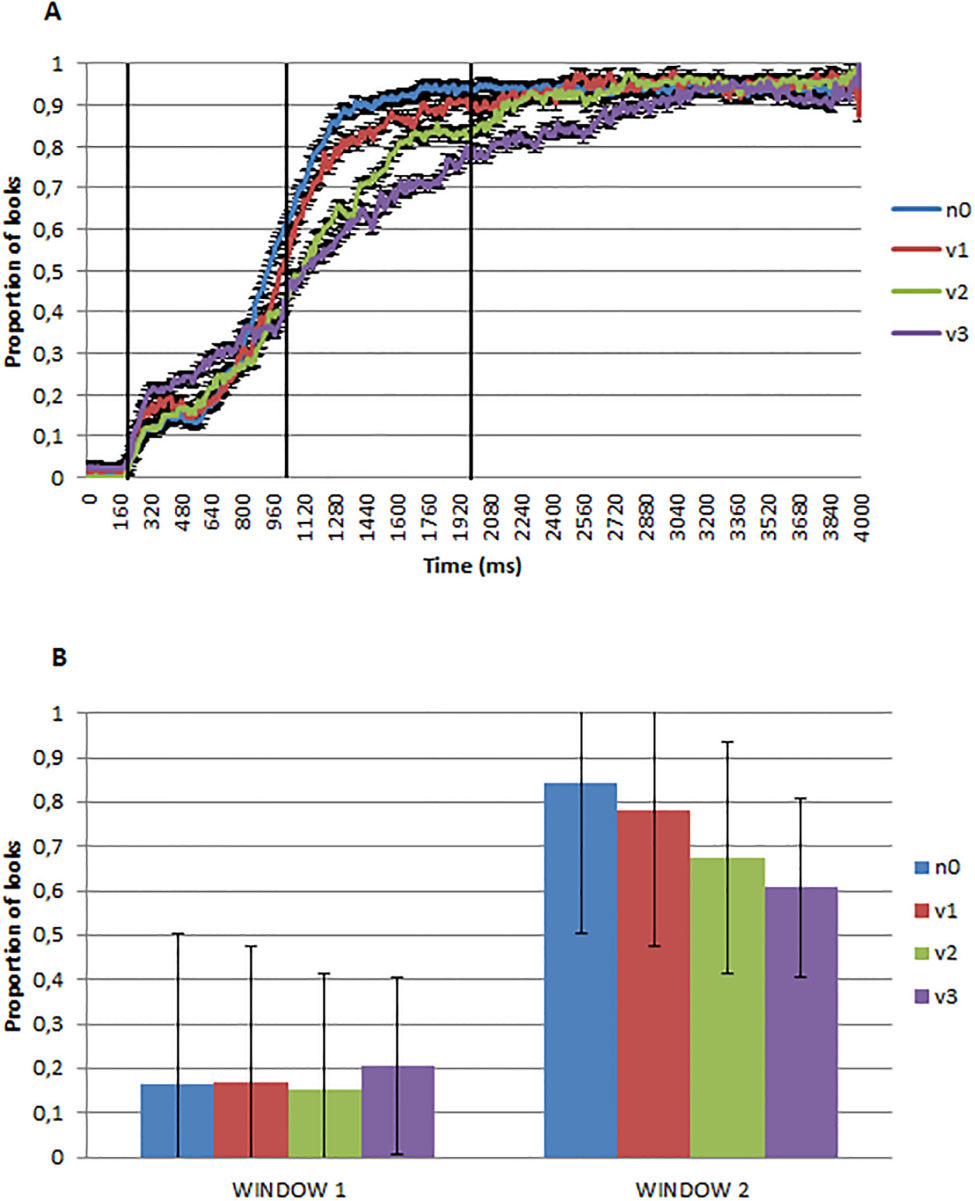
A) Proportion of looks to the filler nouns (n0), one-argument (v1), two-argument (v2) and three-argument target verbs (v3) from image and word onset. B) The same data binned into the two time windows (average of subject means).

Fig 2a presents the proportion of looks over time to the target referent, plotted by time. The three black vertical lines divide the two windows of analysis. Fig 2b presents the same data binned into the two time windows, as defined in the analysis section above.

As shown in Fig 2b, differences between the word types emerge during the second time window. There appear to be effects of verb argument number, such that one argument verbs have more target looks than two argument verbs, which in turn have more looks than three argument verbs. Moreover, as expected, participants were better at finding the nouns as compared to a verb. As shown in the proportion curves in Fig 2a, these differences between conditions emerge toward the end of the first time window, and reflect the speed at which participants can locate the target picture (i.e., they reflect how quickly the proportion curves reached their asymptote of approximately 0.95).

Which of these differences in the second window can be explained as arising from differences in imageability, and which can be associated with verb argument complexity? Recall from the stimuli section that the three verb subsets (1, 2 vs. 3 arguments) did not differ between themselves in terms of imageability/label-appropriateness dimensions, yet target looking times do differ between these verb types. This pattern in the norms suggests, albeit indirectly, that differences among verbs may reflect something about the complexity of the semantics associated with these verbs, rather than imageability/label-appropriateness. However, the filler nouns were rated as being more imageable terms than verbs, and were also rated as being more appropriate labels for their pictures. Thus, it is possible that the speeded identification of nouns over verbs may be related to the syntactic category of the labels or to the fact that the nouns were more imageable and better labels of their pictures than verbs.

**Fig 3:**
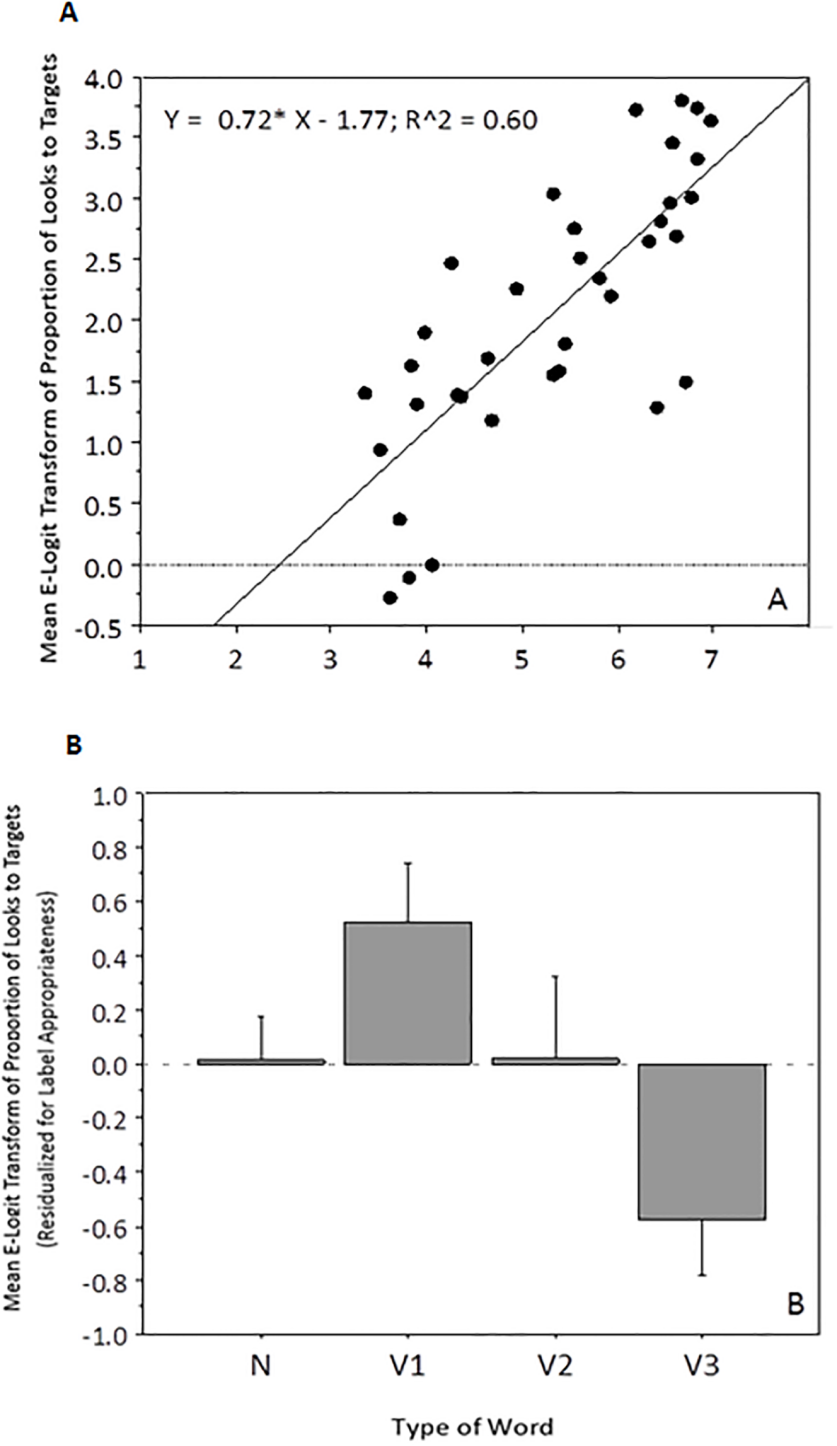
A) Correlation between proportion of time on target verbs and filler nouns and label appropriateness rating. B) Residualized item means by condition (error bars = 1 S.E).

The mean label-appropriateness ratings for each word (regardless of whether it is a verb or a noun) were found to be highly correlated with each item’s mean proportion of target looks during time window 2 (see Fig 3a); people are better able to locate the target picture if the word being uttered is a highly appropriate label for that picture (imagebility correlated with label appropriateness, R^2^ = 0.324; p<0.001, and generates similar results when related to looking times). We therefore focus our discussion on label appropriateness. One can partial out the variance associated with label appropriateness by transforming target item means into residuals, i.e., positive and negative deviations from the fitted line in Fig 3a. Fig 3b plots residualized item means by condition. As can be seen in the Fig 3, nouns were no longer different from verbs as a whole, but verbs show an effect of argument number; one argument events are located faster than what is predicted based on label appropriateness, whereas three argument events are located slower than expected. Simple transitive (two-argument) events and nouns are located just as fast as predicted by label appropriateness alone.

These observations are however based on aggregated data (i.e., item means), and as such may be failing to capture relevant variation [59]. A better way to analyze this data is via multi-level mixed linear modeling of non-aggregated trial-level observations for the verb data only. In particular, the E-logit-transformed proportion of target looks for each trial was modeled using the lmer function in R, with crossed-random intercepts supplied for each Subject and Item. We can enter both argument number (1, 2 vs 3 arguments) and label appropriateness norms (a continuous variable) as predictors, to see how much variance is accounted for by both variables separately and simultaneously. The best fitting model is one that includes a reliable effect of argument number and label appropriateness, both as continuous variables (see Table 3). Thus, argument number and label appropriateness account for different aspects of the variance within verbs.

**Table 3:** Fixed effects from best fitting multi-level linear model of the proportion of target looks, E-logit transformed, time window 2 (Experiment 1).

Effect	Estimate	S.E.	t-value
Intercept	64.55	17.49	3.69*
Label Appropriateness	8.27	3.42	2.41*
Argument Number (1, 2, 3)	-9.27	3.07	-3.02*

* p<0.05 (on normal distribution)

Note: This model had a significantly better fit than an empty model with no fixed effects, based on a chi-square test of the change in quasi-log likelihood [60]. The quasi-log likelihood of the present model (—2862) was a reliably better a fit than a model that contained just label appropriateness (-2866, p<0.01). It was also a reliably better fit than a model that contained just argument number (-2865, p<.06). Neither a model that included the interaction term or word frequency were better fits

In a separate analysis that included both the noun and the verb data, we examined the effect syntactic category (nouns vs. verbs) and label appropriateness norms (a continuous variable) as predictors, to see how much variance is accounted for by both variables separately and simultaneously. Although a model that contained only syntactic category (noun vs. verb) showed a significant effect of this factor (beta estimate = -24.4, t(1) = -4.71, p<0.01), the best fitting model was one that used both label appropriateness and syntactic category as predictors, in which label appropriateness was the only significant predictor (see Table 4). This implies that the variance is better explained by label appropriateness rather than the syntactic category of the label.

**Table 4:** Fixed effects from best fitting multi-level linear model of the proportion of target looks, E-logit transformed, time window 2 (Experiment 1).

Effect	Estimate	S.E.	t-value
Intercept	41.49	20.70	2.00*
Label Appropriateness	11.15	3.25	-3.55*
Syntactic Category (N vs. V)	-3.32	7.63	-0.44

* p<0.05 (on normal distribution)

Note: This model had a significantly better fit than an empty model with no fixed effects, based on a chi-square test of the change in quasi-log likelihood [60]. The quasi-log likelihood of the present model (-5759) was no better a fit than a model that contained just label appropriateness (-5760). It was however a better fit than a model that contained just syntactic category (-5765, p<0.01). Neither a model that included the interaction term or word frequency were better fits.

### Discussion

In this experiment, we observed that there is a reliable linear effect of argument number above any effect of label appropriateness. One Argument (intransitive) events were faster to locate than would be expected given their label appropriateness, whereas three argument (di-transitive) events were slower to locate than would be expected given their label appropriateness; two argument (transitive) events fell in between and were located at a rate expected given their label appropriateness. Explaining this effect as being related to verb argument complexity runs into trouble however because intransitive verbs were also found to be processed more quickly than simple nouns (after factoring out label appropriateness), requiring one to conclude that intransitive verbs are, for some unknown reason, representationally simpler than nouns.

Moreover, we observed that nouns are faster to identify than verbs. However, this effect is carried entirely by the degree to which nouns are better labels for pictures than verbs: Nouns are more imageable than verbs. This relationship has been observed before in a very different experimental setting, in which participants were asked to learn the meanings of nouns and verbs directly from visual observation of the world (see [55]). In Gillette et al. [55], although nouns were found to be learned more easily than verbs by adults (an effect also observed in infants learning their first language), the effect was attributable solely to the imageability of the words, not their syntactic status as a noun or a verb. They concluded that verbs are more difficult to learn from direct observation with the world because verbs are more likely to label aspects of the world that are difficult to see. The present finding offers support for this conclusion, it is harder to locate pictures labeled by verbs as compared to pictures labeled by nouns because verbs are less imageable than nouns.

Experiment 1 has shown that eye movements reveal the time course of the dynamics of lexical activation which improves our measure of lexical processing from previous studies. As we can see in Fig 2, the slope of the curve reflects the speed at which participants can locate the target picture and when the curve reached their asymptote was the moment that the vast majority of participants decided which picture was the target. However, in the present experiment participants were not asked to indicate exactly when they had located the target (e.g., by pressing a button). Instead, they were asked to hold gaze on the target picture. In Experiment 2, we collect button pressing data as an explicit indication of the timing of participants’ decision making.

## Experiment 2

In this second experiment we seek to replicate these observed effects but make alterations to the experiment that might improve our measure of lexical processing time. We used reaction time data collected from button presses. Eye movements were also collected, to examine how this process unfolds over time.

### Method

This study was approved by the Ethics Committee of the Universitat Oberta de Catalunya.

### Participants

Fourteen native Spanish speakers participated in the experiment (8 females and 6 males). All participants were born in Spain and studied primary and secondary school in Spain. They were students or junior faculty at various universities in the Philadelphia area. All gave their written informed consent for participation in this study. They either had uncorrected vision or wore soft contact lenses or eyeglasses.

### Stimuli

The stimuli were the same as those used in experiment 1.

### Procedure

The procedure was the same as experiment 1 except for minor modifications. In particular, participants were instructed to press the spacebar on the computer keyboard as soon as they found the target picture. Pressing the spacebar ended the presentation of the image and caused the presentation of the crosshair for the next trial. Response time from image and word onset was calculated. The equipment and software were also different from Experiment 1; here a *Tobii 1750* eye tracker was used (which has 50 Hz eye sampling rate) and *E-Prime* Software was used to present the stimuli and collect the data. This procedure was approved by the Human Subject panel of the Institutional Review Board (IRB) at the University of Pennsylvania.

### Results

**Fig 4:**
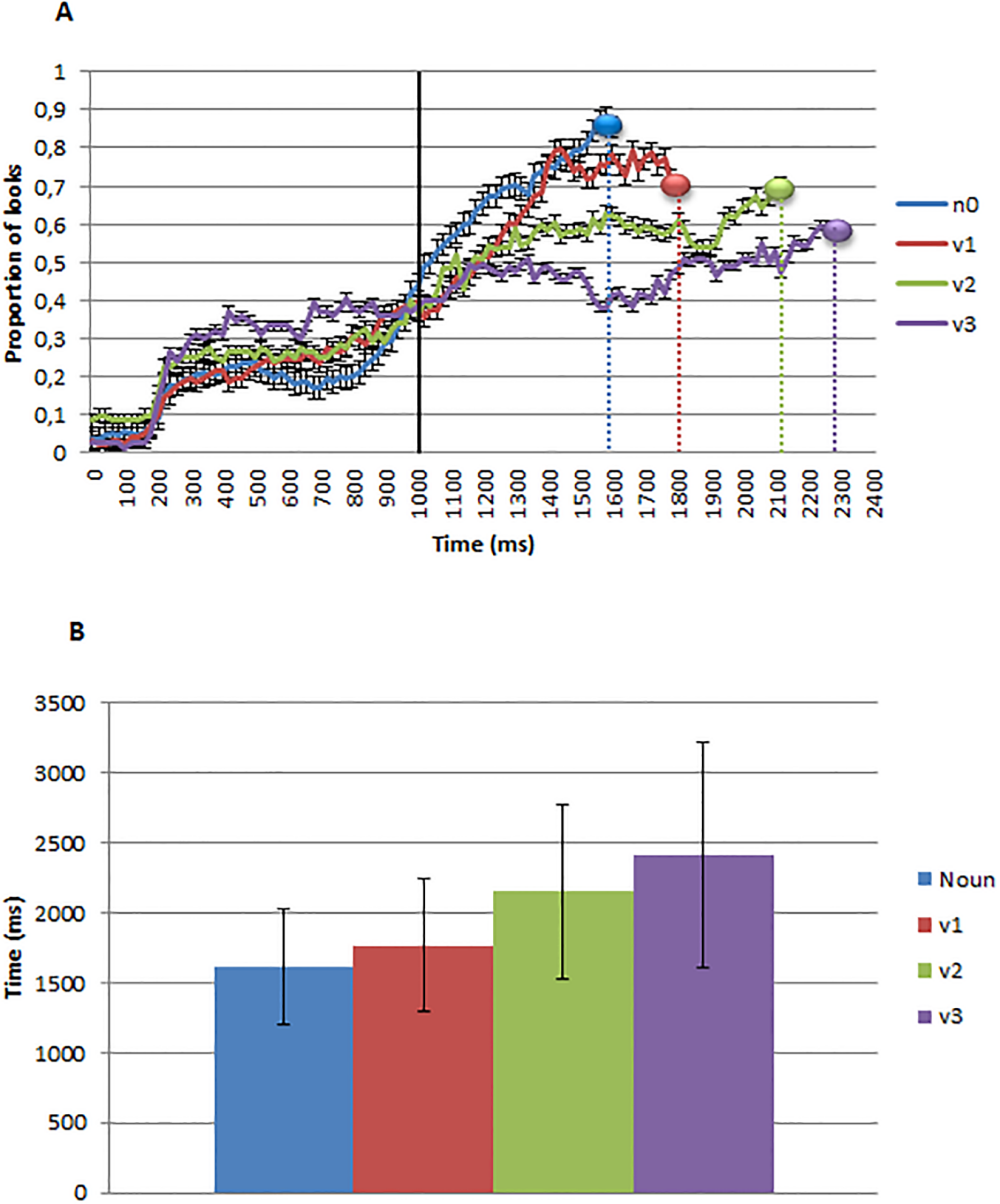
A) Eye tracking data. Proportion of looks to nouns (n0), one-argument (v1), two-argument (v2) and three-argument verbs (v3) from image and word onset. The three dotted vertical lines indicate mean response time for each word type to press the spacebar. B) Reaction time data. Mean response time for each word type to press the spacebar.

Fig 4A presents the proportion of looks over time to the target referent, plotted sample by sample. The single solid vertical line indicates the offset of the word (around 1 second). The four dotted vertical lines indicate mean response time for each word type. As can be seen in Fig 4A and 4B, mean response times conform to what was observed in the eye movements of experiment 1; on average, there was a systematic delay in response time as a function of number of arguments and nouns were identified more quickly than verbs. Eye movement proportions do not converge to 1.0 because as expected from a normal distribution of response times, approximately half of the participants had yet to locate the target by the time the mean response time had been reached.

**Fig 5:**
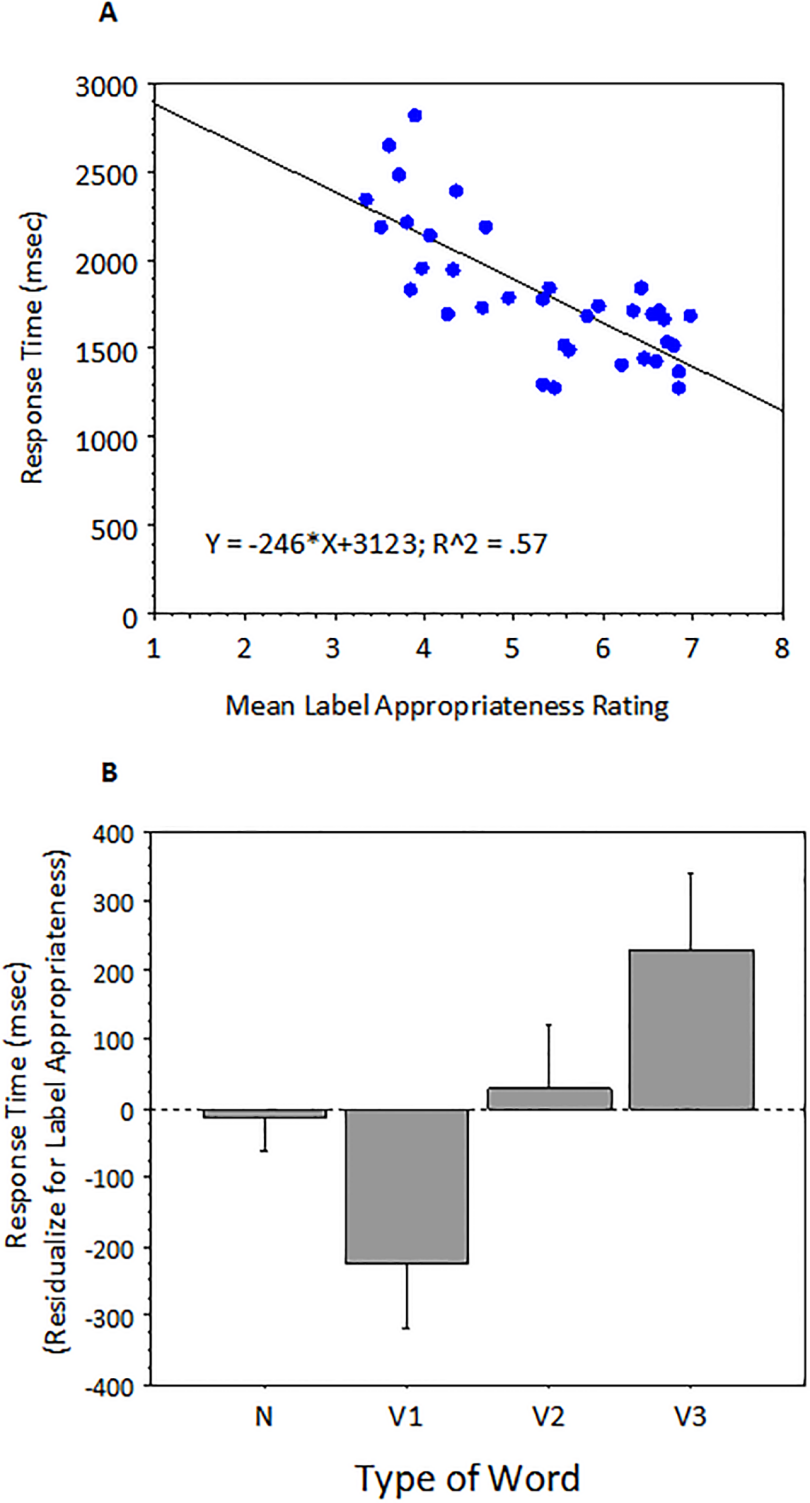
A) Correlation between proportion of time on target verbs and filler nouns and label appropriateness rating. B) Residualized item means by condition (Error Bars = 1 S.E).

Like experiment 1, our primary dependent measure (here, reaction time) correlated with label appropriateness (see Fig 5A). In terms of item means, words that were rated as being better labels for their pictures resulted in faster response times. Following the data analysis from experiment 1, we can partial out the variance due to label appropriateness by plotting reaction time in terms of residual response times. This is presented in Fig 5B, for each of the four word types.

Following what was done in experiment 1, we modeled response time using multi-level mixed linear modeling of non-aggregated trial-level observations. Response time (in msec) for each trial was modeled using the lmer function in R, with crossed-random intercepts supplied for each subject and item. Argument number (1, 2 vs 3 arguments) and label appropriateness norms (a continuous variable) were used as predictors. The best fitting model was one that included a reliable effect of argument number and Label appropriateness, both as continuous variables (see Table 5). Thus, like Experiment 1, argument number and label appropriateness accounted for different aspects of the variance within verbs.

**Table 5:** Fixed effects from best fitting multi-level linear model of the response time in milliseconds (Experiment 2).

Effect	Estimate	S.E.	t-value
Intercept	2906	411	7.07*
Label Appropriateness	-295	78	-3.77*
Argument Number (1, 2, 3)	219	70	3.12*

* p<0.05 (on normal distribution)

Note: This model had a significantly better fit than an empty model with no fixed effects, based on a chi-square test of the change in quasi-log likelihood [60]. The quasi-log likelihood of the present model (—1933) was a reliably better a fit than a model that contained just argument number (-3906, p<0.001). It was also a reliably better fit than a model that contained just label appropriateness (-3903, p<.01). Neither a model that included the interaction term or word frequency were better fits.

Moreover, as in experiment 1 we also used syntactic category (nouns vs. verbs) and label appropriateness norms (a continuous variable) as predictors. Although a model that contained only syntactic category (noun vs. verb) showed a significant effect of this factor (B = -24.4, t(1) = -4.71, p<0.01), the best fitting model was one that also used label appropriateness as a predictor, resulting in no reliable effect of syntactic category (see Table 6). This implies that the variance is better explained by Label Appropriateness rather than the syntactic category of the label.

**Table 6:** Fixed effects from best fitting multi-level linear model of the response time in milliseconds (Experiment 2).

Effect	Estimate	S.E.	t-value
Intercept	2963	405	7.30*
Label Appropriateness	-223	63	-3.55*
Syntactic Category (N vs. V)	-68	147	0.46

* p<0.05 (on normal distribution)

Note: This model had a significantly better fit than an empty model with no fixed effects, based on a chi-square test of the change in quasi-log likelihood [60]. The quasi-log likelihood of the present model (-3779) was no better a fit than a model that contained just label appropriateness (-3779). It was however a better fit than a model that contained just syntactic category (-3785, p<0.01). Neither a model that included the interaction term or word frequency were better fits.

### Discussion

The eye movement effects observed in experiment 1 were also observed here using a different dependent measure: response time. There was a reliable linear effect of argument number above any effect of label appropriateness, just like experiment 1. One argument (intransitive) events were faster to locate than would be expected given their label appropriateness, whereas three argument (di-transitive) events were slower to locate than would be expected given their label appropriateness; two argument (transitive) events fell in between and were located at a rate expected given their label appropriateness. Moreover, as expected, nouns were faster to identify than verbs and this effect was carried by the degree to which nouns were better labels for pictures than verbs.

However, one limitation of both experiments is that we did not control the complexity of the images. Visual complexity contributes to difficulty in picture decoding [61] and then can affect the time to recognition. Moreover, there are more variables that can affect the auditory word recognition that we did not control, such as oral lexical frequency or phonological neighborhood.

## Experiment 3

Based on the limitations set in the discussion of experiment 2, we ran a third experiment in which we controlled more variables for the stimuli selection. We controlled the visual complexity and other variables that affect the auditory word recognition (phoneme length, oral lexical frequency, phonological neighborhood, etc.). Moreover, we extended the sample and the number of verb stimuli. In experiments 1 and 2, we only had six verbs of each type (one-, two-, three- arguments). Here we selected eighteen of each verb type and increased the number of participants to get more robust results.

### Method

This study was approved by the Ethics Committee of the Universitat Oberta de Catalunya.

### Participants

Ninety-five participants took part in the experiment. All participants were born in Spain and studied primary and secondary school in Spain. They were all native Spanish speakers studying the degree of Psychology at the University of Barcelona with normal or corrected-to normal vision. They participated in the experiment in exchange for course credits. All the participants gave their written informed consent for participation in this study.

### Stimuli

The stimuli included 54 verbs, 18 one-argument, 18 two-argument and 18 three-argument verbs (see S1 Appendix). A list of 18 Nouns was selected as fillers. Argument structure was determined by a search in a syntactic database of Spanish usage [62]. The three subsets of verbs, plus the nouns, were matched on letter, phoneme and syllable length, as well as phonological neighborhood [63], and oral [64] and written [52] lexical frequencies. Stimuli words were also controlled for imageability [53] and rated age of acquisition [53]. When values of any of these two variables for any of the stimuli were missing in the databases, specific surveys were carried out following the same guidelines used in the original studies. Groups of 25 raters, different from the volunteers participating in the experiments, answered the surveys.

Each word stimulus was paired with a picture showing the intended action or object. Like in the previous experiments, name-image appropriateness data were gathered. The same procedure used in the previous experiments was applied. In this experiment, however, a name agreement survey was also included. A group of 20 volunteers were asked to name the experimental pictures. Then percentages of agreement were calculated. The three verb subsets were matched on both label appropriateness and name agreement (see Table 7).

**Table 7:** Summary of stimuli characteristics for experiment 3, SDs and ranges appear in parentheses.

	One-argument N = 18 Mean (S.D.) (range)	Two-argument N = 18 Mean (S.D.) (range)	Three-argument N = 18 Mean (S.D.) (range)	Filler nouns N = 18 Mean (S.D.) (range)
*Letters*	5.83 (1.34) (3–8)	5.5 (1.82) (0–9)	5.72 (0.89) (4–8)	5.5 (1.5) (3–9)
*Phonemes*	5.67 (1.19) (3–8)	5.72 (1.13) (4–8)	5.56 (0.92) (4–8)	5.33 (1.37) (3–8)
*Syllables*	2.22 (0.55) (1–3)	2.22 (0.43) (2–3)	2.17 (0.38) (2–3)	2.5 (0.71) (2–4)
*Phonological Neighborhood*	13.3 (6.9) (5–30)	15.3 (7.7) (4–29)	16.3 (7.6) (5–30)	16.8 (13.5) (1–49)
*Written Frequency*	35.86 (62.2) (0.18–249.11)	19.05 (18.76) (0.71–70.54)	15.68 (18.19) (1.61–60.54)	94.62 (340.73) (0–1458.75)
*Oral Frequency*	38.96 (52.3) (1–191.16)	24.11 (25.11) (1.42–74.12)	22.82 (41.77) (0–173.08)	77.86 (263.35) (0.29–1130.87)
*Age of Acquisition*	2.98 (0.64) (1.8–4.12)	3.05 (0.7) (1.83–4.88)	2.83 (0.61) (1.76–3.96)	2.7 (0.48) (1.92–3.5)
*Imageability*	5 (0.83) (3.64–6.36)	5.39 (0.58) (4.14–6.3)	5.48 (0.62) (4.61–6.38)	5.43 (0.31) (4.62–5.79)
*Visual Complexity*	38.4 (5.77) (26.92–46.26)	36.17 (9.22) (25.98–61.6)	33.86 (12.38) (24.45–80.8)	33.55 (17.94) (18.93–76.2)
*Label Appropriateness*	5.87 (0.67)* (4.81–6.81)	6.05 (0.6)* (4.81–6.9)	6.13 (0.6)* (4.67–6.95)	6.67 (0.36) (5.86–7)
*Name Agreement*	67.46 (25.75)* (25–100)	82.5 (22.51) (20–100)	83.57 (21.85) (26.32–100)	91.53 (7.58) (71.43–95.24)

Note: Letters: number of letters; phonemes: number of phonemes; syllables: number of syllables; phonological neighborhood: number of substitution, addition, and deletion phonological neighbors; written frequency: word frequency per million; oral frequency: word frequency per million; age of acquisition: mean subjective age of acquisition in a 1–7 scale; imageability: mean subjective imageability in a 1–7 scale; visual complexity: JPEG compression file sizes in KB; label appropriateness: subjective appropriateness ratings in a 1–7 scale; name agreement: percentage of label coincidences.

* Significantly different to the noun set at p<.05

Following Rodríguez-Ferreiro et al. [65], values for the visual complexity of the pictures were obtained using the JPEG compression method described in Bates et al. [66]. All the images were compressed in the Joint Photographic Experts Group (JPEG) format and the size of the digitized picture file was used as a value of visual complexity. This method provides an objective measure of how complex a picture is, avoiding the confounds with other variables that appear when subjective ratings are used [67, 68]. For example, Székely and Bates [68] have shown that subjective ratings of complexity are confounded with subjective judgments of familiarity. Intransitive verbs and filler nouns appeared to be associated with less visually complex scenarios. In order to eliminate significant differences between these and the transitive and ditransitive items, background textures were included in some of their corresponding pictures until equal visual complexity values were obtained in all the stimuli sets.

Although the distracter pictures were never referred to with names during the experiment, we made sure that what we considered to be common names for these distracter pictures were similar in the frequency of written Spanish to the target names using the LEXESP corpus [52]. In addition, the onset phoneme of each target word always differed from the onset phoneme of the common names for the three distracters, so to avoid auditory cohort competitor effects (see [5]).

A presentation list was created such that each verb target picture was paired with three pictures resulting in a set of four images that always included two object images and two event images. Then, targets had one event competitor and two object distracters. A second and third list was generated from the first list by changing the competitor’s event images. So, then each target verb had as competitor an event of each verb type (one-, two- and three-argument verb).

The visual and auditory stimuli were created by the same procedure that experiments 1 and 2.

### Procedure

The procedure was the same as experiment 2 except for the equipment and software that was the same that experiment 1.

### Results

**Fig 6:**
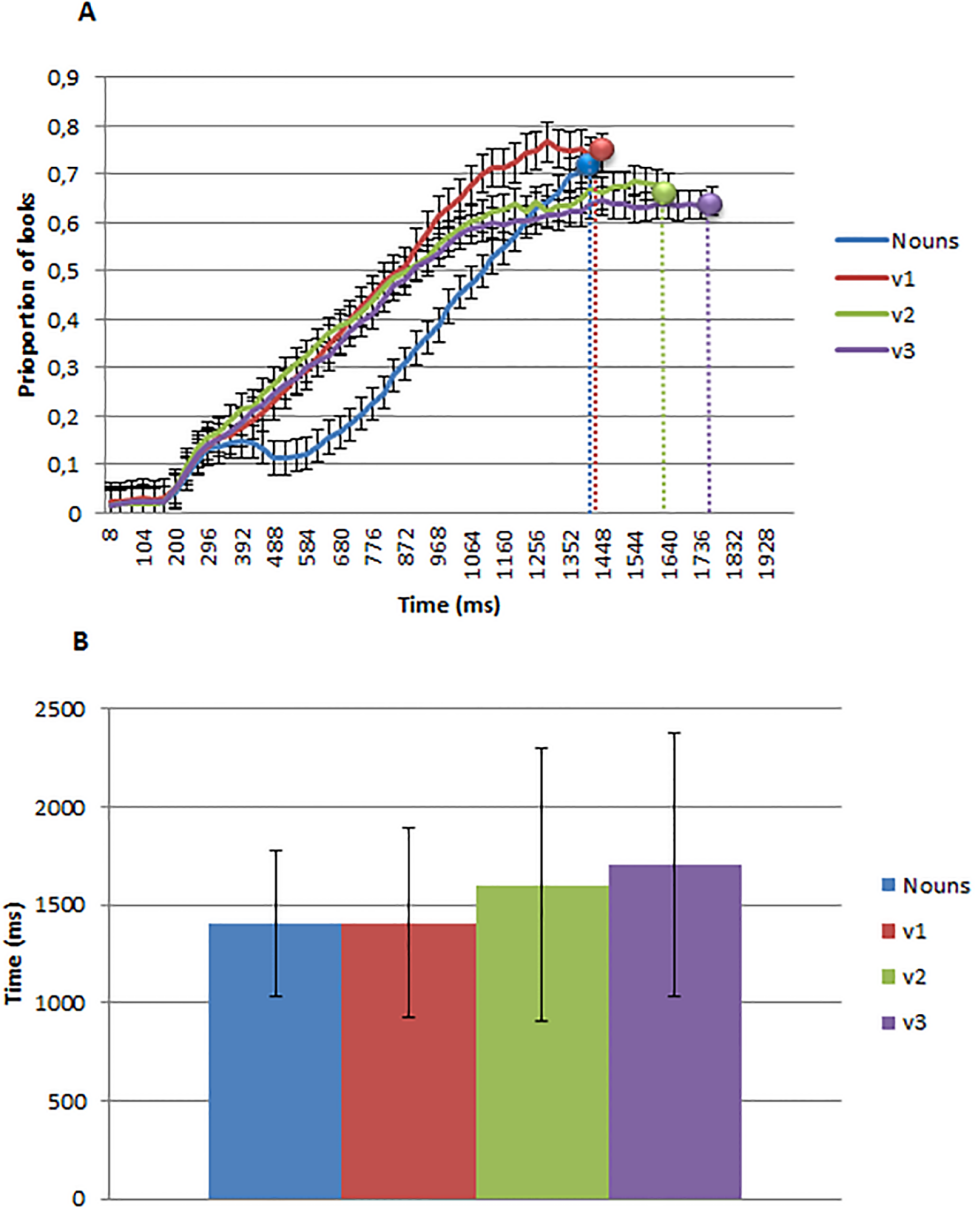
A) Eye tracking data. Proportion of looks to nouns (n0), one-argument (v1), two-argument (v2) and three-argument verbs (v3) from image and word onset. The three dotted vertical lines indicate mean response time for each word type to press the spacebar. B) Reaction time data. Mean response time for each word type to press the spacebar.

Fig 6A presents the proportion of looks over time to the Target referent, plotted sample by sample. The three dotted vertical lines indicate mean response time for each word type. As can be seen in the Fig 6A and 6B, mean response times differ somewhat from previous experiments. On average, response time increased as a function of Number of Arguments.

Following the analyses procedure used in experiment 2, we used multi-level mixed linear modeling of non-aggregated trial-level observations to model the participants’ reaction times. Latency data was first log (base 10)-transformed to prevent spurious influence of the marked skew associated with chronometric data [69]. We report Markov chain Monte Carlo (MCMC)-derived p values for effects following Baayen [70]. A first model including the verb type as independent variable showed an effect of the continuous variable argument number (t(1) = 3.21, pMCMC<.001). Post-hoc TukeyHSD contrasts showed significant differences in the pairwise comparison between transitive and intransitive verbs, transitive and ditransitive verbs, and between intransitive and ditransitive verbs (all ps<.001). The effect of argument number continued to be significant (t(1) = 2.91, pMCMC<.001) when we tested a model also including Label Appropriateness (t(1) = -3.25, pMCMC<.001).

Moreover, using syntactic category (noun vs. verb) as the independent variable we found a significant effect of this factor (t(1) = 1.72, pMCMC = 0.02). However, this effect disappeared (t(1) = 0.10, pMCMC = 0.87) when we introduced the variable label appropriateness, which was significant (t(1) = -3.32, pMCMC<.001).

### Discussion

In this experiment we controlled other variables than in previous experiments (number of letters, phonemes, syllables; phonological neighborhood; written frequency, oral frequency, age of acquisition, imageability and visual complexity). We increased the number of stimuli (54 verbs, 18 one-argument, 18 two-argument and 18 three-argument verbs and 18 filler nouns) and the number of participants (ninety-five adults). The response time effects observed in Experiment 2 were also observed here. We found that there is a reliable linear effect of argument number above any effect of label appropriateness. Nouns were faster to identify than verbs and this effect was carried entirely by the degree to which nouns are better labels for pictures than verbs.

In this experiment we controlled visual complexity. However, it should be noted that in this experiment and the previous experiments, the number of people depicted in each picture differed systematically with verb type. It is therefore possible that regardless of the visual complexity of the scenes, listeners take longer to infer the relationships between two event participants and an object (ditransitive verbs) than if a single event participant is acting alone. Perhaps, having bystanders in the scene might help to alleviate this concern.

## General discussion

In three experiments of picture identification in response to a spoken word, it was observed that the number of arguments a verb takes, impacted negatively on target identification times, the greater the number of arguments, the slower the response time. The timing of eye movements to the target image also supported this conclusion.

Previous work has found inconsistent results relating lexical processing time to verb argument complexity. On the one hand, Shapiro and collaborators [35–37] showed that verb’s representational complexity (syntactic subcategorization and argument structure) affects real-time sentence processing. On the other hand, Schmauder and collaborators [38–39] did not find this effect in cross-modal lexical decision and monosyllabic secondary lexical decision tasks. Our results replicate the finding of Shapiro and collaborators [35–37] but using the *visual world paradigm*. This paradigm allowed us to analyse the recognition time that participants need after listening to an isolated auditory verb. Our results cannot explain fully the inconsistent findings that exist in the past literature on this topic. It is notable though that much of the past work used the subjects’ performance in an unrelated secondary task as a measure of processing difficulty with the primary task of language comprehension. It is possible that such indirect measures, especially in a dual-task paradigm, produce inconsistent results. Our own work used what is arguably a more direct measure of processing difficulty (response time), embedded within a relatively natural task in which subjects attempt to link speech to a co-present referent world. This paradigm may make it easier to identify consistent results of verb argument complexity.

As we see it, there are two explanations of our verb-argument findings. One possible explanation of our results is that differences in sheer representational complexity between these three classes of verbs explain this effect in response time. Previous studies have proposed this hypothesis (e.g., [33, 34, 44]). However, the results from our noun items draws this conclusion into question. Although we found that nouns are recognized more quickly than verbs, this difference was attributable to imageability and the degree to which the word was a good label for the target picture. After removing imagability factors, nouns were no easier to process than verbs, despite the fact that (most) verbs have more complex representations. This findings suggests that the hypothesized greater complexity of the information associated with verbs as compared to nouns does not negatively impact lexical processing, in line with processing theories that do not link the sheer amount of information activated with the amount of processing time needed (e.g., [40–42]).

Another possibility is these differences between verbs are related to how much the information generated by the lexical item aids the task that was given to the participant: target image identification. For example, each argument of a verb has a range of possible participants associated with it. “Sleeping” (an intransitive event) can be done by a human or by an animal, such as a dog, cat, etc. Likewise “licking” (a transitive event) can be done by a human or an animal, yet now what is licked can vary (a lollipop, a popsicle, etc.). Ditransitive events, such as “throwing” have similar uncertainties as transitive verbs, yet in addition, the recipient is uncertain (for instance, one can throw something to a human or to an animal). If listeners in this task are computing a mental image based on the semantics activated by the lexical item and then are seeking out an actual image that matches that mental image (an assumption that has some experimental support, see Dahan and Tanenhaus [71]), then one would expect the increases observed here within verbs: each additional argument increases the chances of image mismatches. However, like the first account, this account would need to explain why nouns do not show a significant advantage over verbs.

Future research is needed to analyse in greater detail if the recognition times between the different verb types are due to verb argument complexity or to the knowledge that is activated to accomplish the task. The present work therefore offers an additional step toward understanding the role of representational complexity in language comprehension. We believe that direct comparison of methodologies and tasks will likely offer further illumination of this issue.

## Supporting information

S1 Appendix List of verbs used as stimuli for experiment 1, 2 and 3. (DOCX)

S2 Appendix Eye tracking data from the 3 experiments. (XLSX)
