# Supplementary material for: Auditory word recognition of verbs: Effects of verb argument structure on referent identification
Source: PLoS One. 2017 Dec 5;12(12):e0188728. doi: 10.1371/journal.pone.0188728 (PMC5716596; doi:10.1371/journal.pone.0188728)
Supplement: S1 Appendix — (DOCX) [file pone.0188728.s001.docx]

**Appendix A**

List of target verbs and filler nouns used as stimuli in experiment 1 and 2.

| ***One-argument verbs*** | ***Two-argument verbs*** | ***Three-argument verbs*** | ***Nouns*** |  |
| --- | --- | --- | --- | --- |
| Bailar  *[Dance]* | Abrir  *[Open]* | Atar  *[Tie]* | Árbol *[Tree]* |  |
| Caer  *[Fall]* | Coger  *[Catch]* | Dar  *[Give]* | Avión *[Plane]* |  |
| Caminar  *[Walk]* | Chupar  *[Lick]* | Enseñar  *[Show]* | Cama *[Bed]* |  |
| Dormir  *[Sleep]* | Llevar  *[Carry]* | Regalar  *[Give (a present)]* | Coche *[Car]* |  |
| Llorar  *[Cry]* | Recoger  *[Pick]* | Romper  *[Break]* | Flor *[Flower]* |  |
| Volar  *[Fly]* | Tocar  *[Play]* | Tirar  *[Throw]* | Lámpara *[Lamp]* |  |
|  |  |  | Lápiz *[Pencil]* |  |
|  |  |  | Llave *[Key]* |  |
|  |  |  | Manzana *[Apple]* |  |
|  |  |  | Mesa *[Table]* |  |
|  |  |  | Muñeco *[Doll]* |  |
|  |  |  | Radio *[Radio]* |  |
|  |  |  | Reloj *[Clock]* |  |
|  |  |  | Silla *[Chair]* |  |
|  |  |  | Sofá *[Sofa]* |  |
|  |  |  | Tarta *[Cake]* |  |
|  |  |  | Tomate *[Tomatoe]* |  |
|  |  |  | Vaso *[Glass]* |  |

**Appendix B**

List of verbs used as stimuli for Experiment 3.

| ***One-argument Verbs*** | ***Two-argument Verbs*** | ***Three-argument Verbs*** | ***Filler nouns*** |
| --- | --- | --- | --- |
| Aullar  *[To howl]* | Abrir  *[To open]* | Abrochar  *[To fasten]* | Alcachofa  *[Artichoke]* |
| Bucear  *[To dive]*  Correr  *[To run]*  Dormir  *[To sleep]*  Flotar  *[To float]* | Arreglar  *[To repair]*  Borrar  *[To rub out]*  Botar  *[To bounce]*  Cerrar  *[To close]* | Arrancar  *[To pull out]*  Clavar  *[To stick]*  Contar  *[To tell]*  Dar  *[To give]* | Ancla  *[Anchor]*  Arpa  *[Harp]*  Blusa  *[Blouse]*  Cañón  *[Cannon]* |
| Girar  *[To twirl]*  Gritar  *[To shout]*  Ladrar  *[To bark]*  Llorar  *[To cry]*  Nadar  *[To swim]*  Patinar  *[To skate]*  Reír  *[To laugh]*  Resbalar  *[To slip]* | Coger  *[To catch]*  Cruzar  *[To cross]*  Doblar  *[To fold]*  Empujar  *[To push]*  Encender  *[To switch on]*  Leer  *[To read]*  Marcar  *[To score]*  Pelar  *[To peel]* | Enseñar  *[To show]*  Entregar  *[To deliver]*  Lanzar  *[To throw]*  Lavar  *[To wash]*  Pedir  *[To ask for]*  Pegar  *[To paste]*  Poner  *[To put]*  Quitar  *[To take away]* | Cebra  *[Zebra]*  Corona  *[Crown]*  Escoba  *[Broom]*  Lechuga  *[Lettuce]*  Luna  *[Moon]*  Maleta  *[Suitcase]*  Montaña  *[Mountain]*  Oso  *[Bear]* |
| Rezar  *[To pray]* | Perseguir  *[To chase]* | Regalar  *[To give (a present)]* | Casco  *[Helmet]* |
| Rugir  *[To roar]* | Picar  *[To bite]* | Rizar  *[To curl]* | Regla  *[Ruler]* |
| Soplar  *[To blow]*  Volar  *[To fly]*  Votar  *[To vote]* | Pisar  *[To tread on]*  Romper  *[To break]*  Tocar  *[To play]* | Robar  *[To steal]*  Sacar  *[To pull]*  Secar  *[To dry]* | Semáforo  *[Traffic light]*  Sobre  *[Envelope]*  Taza  *[Cup]* |
